# Intravenous ondansetron for the prevention of supine hypotensive syndrome during spinal anesthesia for cesarean section: a randomized controlled trial

**DOI:** 10.3389/fphar.2024.1194196

**Published:** 2024-01-18

**Authors:** Yuan Zhang, Fen Xiao, Wangping Zhang

**Affiliations:** ^1^ Department of Anesthesia, Shaoxing People’s Hospital, Shaoxing, China; ^2^ Department of Anesthesiology, Jiaxing Women and Children’s Hospital, Jiaxing, China

**Keywords:** ondansetron, supine hypotensive, syndrome, spinal anesthesia, cesarean section

## Abstract

**Background:** Supine hypotensive syndrome is a common complication in late pregnancy. This study aims to explore the effects of ondansetron on the prevention of supine hypotensive syndrome during spinal anesthesia for cesarean section.

**Methods:** A total of 80 women undergoing elective cesarean delivery were randomly assigned to two groups (the ondansetron group and the control group), with 40 cases in each group. The ondansetron group received 0.075 mg/kg of ondansetron intravenously 5 min before the induction of spinal anesthesia; the control group was given the same volume of saline solution. The blood pressure and heart rate were measured. Umbilical artery pH was analyzed, and the incidence of nausea and vomiting and vasoconstrictor drug usage were noted.

**Results:** The incidence of supine hypotensive syndrome, nausea and vomiting, and vasoconstrictor drug use were significantly lower in the ondansetron group than the control group (2.5% vs. 20%, *p* = 0.029; 2.5% vs. 22.5%, *p* = 0.007; and 5% vs. 22.5%, *p* = 0.012, respectively). Umbilical artery pH was higher in the ondansetron group than the control group, and statistical significance was observed (7.31 ± 0.03 vs. 7.28 ± 0.04, *p* = 0.002). The maternal hemodynamic parameters and the neonatal Apgar score were similar between the two groups.

**Conclusion:** Ondansetron can effectively prevent supine hypotensive syndrome, reduce the incidence of nausea, vomiting, and vasoconstrictor drug use, and improve neonatal umbilical arterial pH during spinal anesthesia for cesarean section.

**Clinical Trial Registration:** https://www.chictr.org.cn/, identifier ChiCTR180018756.

## 1 Introduction

Supine hypotensive syndrome is a common complication during the perioperative period. It is characterized by severe hypotension, accompanied by dizziness, chest tightness, and paleness, which is caused when the gravid uterus compresses the inferior vena cava during pregnancy (De-Giorgio et al., 2012; Humphries et al., 2020). Supine hypotensive syndrome can lead to fetal hypoperfusion and affect the health of the mother and infant, so it is very important to take effective measures to prevent and treat supine hypotensive syndrome during cesarean section. Postural intervention is an effective measure for preventing and treating supine hypotensive syndrome during caesarean section, which is done by turning the woman on her side (Kinsella and Lohmann, 1994) In addition, fluid preload and vasoconstrictors can effectively prevent the episode of hypotension after spinal anesthesia (Massoth et al., 2022).

Ondansetron is a highly selective serotonergic antagonist. It is often used to prevent and treat nausea and vomiting caused by cancer chemotherapy and surgery. Literature reports that ondansetron could suppress serotonin-induced vasodilation by reducing the Bezold–Jarisch reflex (Ortiz-Gómez et al., 2014; Ortiz-Gómez et al., 2017). This study aimed at investigating whether ondansetron could prevent supine hypotensive syndrome in cesarean sections under spinal anesthesia.

## 2 Materials and methods

This study was conducted in accordance with the Declaration of Helsinki and approved by the hospital’s Ethics Committee on 6 November 2019. This trial was registered in the Chinese Clinical Trial Registry (registration number: ChiCTR180018756). All parturients signed written informed consent.

From November 2019 to March 2021, a total of 80 term parturient women undergoing elective cesarean section without symptoms of supine hypotension were included in this trial. The inclusion criteria were as follows: singleton, American Society of Anesthesiologists (ASA) physical status I–II, gestational age >37 weeks, height ranged from 153 to 175 cm, and weight ranged between 55 and 90 kg. The exclusion criteria included pregnancy-related hypertensive disease, cerebrovascular disease, and any spinal anesthesia contraindications. The parturients were randomly divided into the ondansetron and control groups using a computer-generated random number code. The surgeons, anesthetists, and investigators were blinded to the group allocation.

No premedication was given to the parturients. After entering the operating center, the vital signs, including the electrocardiogram (ECG), non-invasive blood pressure (BP), pulse oxygen saturation (SpO_2_), and heart rate (HR), were monitored using an anesthesia monitor. The hemodynamic parameters were measured at 2-min intervals. The baseline values of BP and HR were calculated as the means of three successive measurements. Subsequently, an 18-gauge intravenous cannula was inserted into the vein in the forearm. Lactated Ringer’s solution was infused at a rate of 10 mL kg^−1^·h^−1^ until delivery. Thereafter, it was infused at a rate of 5 mL·kg^−1^ h^−1^.

The ondansetron group received 0.075 mg/kg ondansetron intravenously 5 min before the induction of spinal anesthesia, and the control group received an equal volume of normal saline solution. These solutions were prepared by nurses who were not involved in this study. Anesthesia puncture was carried out in the left lateral decubitus position. At the estimated L3 to L4 vertebral interspace, an 18-gauge Tuohy needle was inserted into the epidural space, and a 25-gauge spinal needle was inserted into the subarachnoid space via the Tuohy needle. After free cerebrospinal fluid flow was observed, a mixture of 0.5% hyperbaric bupivacaine 10 mg and sufentanil 2.5 μg (2 mL in total) was administered intrathecally over 10 s with the needle orifice facing cephalad, and an epidural catheter was inserted 3–4 cm cephalad into the epidural space. The parturients were immediately returned to the supine position with a 10-degree tilt to the left side after spinal anesthesia. The systolic blood pressure (SBP), diastolic blood pressure (DBP), and HR were noted at the following time points: 5 min before spinal anesthesia induction (T0), 5 min after spinal anesthesia induction (T1), at skin incision (T2), and delivery (T3). Vasoconstrictors were administered for the following indications: *a*. mean arterial pressure <60 mmHg; *b*. nausea and vomiting; *c*. stuffiness and palpitation or dizziness; and *d*. patients complained of discomfort as hypotension or hypotension-related discomfort. A rescue bolus dose of phenylephrine 50 µg was given when any of the above conditions occurred. Atropine 0.3–0.5 mg was administered intravenously when HR was <50 beats/min. After a bilateral T6 sensory block was achieved, a surgical incision was allowed. After delivery, umbilical artery blood was drawn for analysis, 5 U of oxytocin was administered muscularly *in utero*, and 3 U of oxytocin was administered by intravenous bolus.

The Apgar scores of the newborns at 1 and 5 min and the pH of the umbilical artery were evaluated. The incidence of maternal hypotension, bradycardia, nausea and vomiting, and vasoconstrictor drug use were noted during spinal anesthesia. Maternal hypotension was defined as an SBP <80% of baseline. Bradycardia was defined as HR < 60 beats/min. Supine hypotensive syndrome was defined as severe hypotension (SBP drop by more than 30% of baseline), accompanied by dizziness, chest tightness, and paleness. Vasoconstrictor drugs were used when supine hypotensive syndrome occurred or patients complained of nausea and vomiting, had an SBP below 70% of baseline, or showed other hypotension-related complications. The maximum sensory block level was measured within the first 10 min after intrathecal administration using a pinprick at 2-min intervals, the induction-to-incision interval, and the induction-to-delivery interval, and the minimal mean arterial pressure was also recorded.

### 2.1 Statistical analysis

The incidence of supine hypotensive syndrome was the primary outcome of this trial. The size of the sample was calculated in accordance with our pilot study. It was estimated that a sample size of 32 women in each group could detect a difference of 60% in the incidence of supine hypotensive syndrome between the two groups before delivery, with an alpha error of 0.05 and a power of 0.8. To account for dropouts, the sample size in each group was increased to 40. The quantitative data with a normal distribution were analyzed using the *t*-test and analysis of variance (ANOVA). Categorical variables were compared using the chi-squared test or Fisher’s exact test. A *p*-value of <0.05 was considered statistically significant.

## 3 Results

**FIGURE 1:**
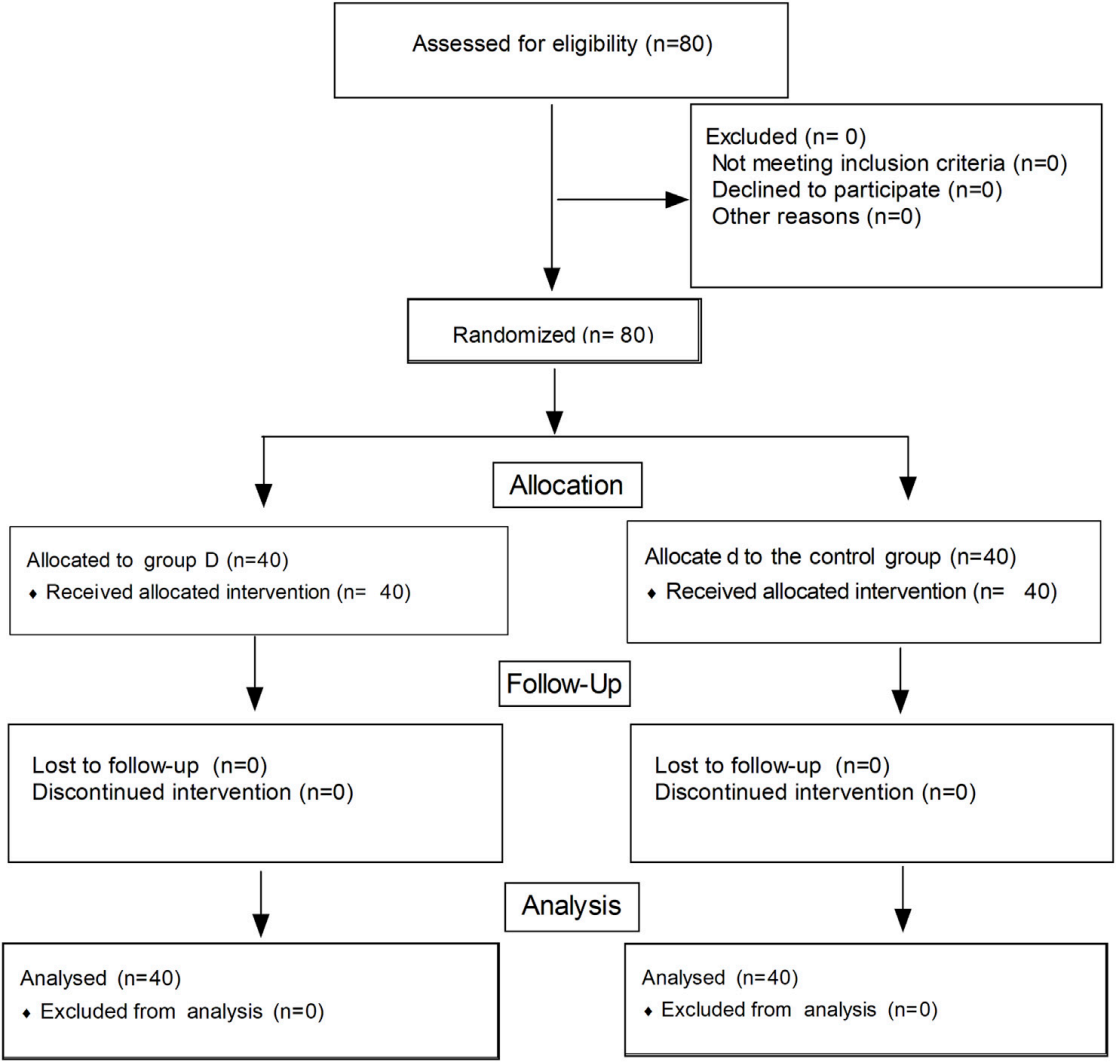
CONSORT 2010 flow diagram.

The flow diagram of this study is shown in Figure 1. A total of 80 women were enrolled in this study and completed the trial. There were no significant differences in terms of maternal age, height, body mass index, and gestational week between the two groups (Table 1). No significant differences were observed in terms of the induction-to-delivery interval, highest levels of sensory block, and total volume of fluid given; there was no statistical significance (*p* > 0.05). The rate of vasoconstrictor usage was significantly lower in the ondansetron group than the control group (5% vs. 22.5%, *p* = 0.012). The minimal mean arterial pressure was higher in the ondansetron group than the control group (71.4 ± 4.9 vs. 68.8 ± 6.4 mmHg, *p* = 0.028). Umbilical artery pH was higher in the ondansetron group than the control group; there was a statistical significance (7.31 ± 0.03 vs. 7.28 ± 0.04, *p* = 0.002), but there were no statistical significances in terms of the Apgar score at 1 min and 5 min between the two groups (*p* > 0.05) (Table 2).

**TABLE 1:** Characteristics of the study population.

Index	Ondansetron group (*n* = 40)	Control group (*n* = 40)	*p*-value
Age (year)	27.2 ± 2.6	27.1 ± 3.2	0.879
Height (cm)	160.8 ± 4.2	159.6 ± 2.9	0.109
Weight (kg)	69.4 ± 6.7	68.7 ± 7.4	0.638
Body mass index (kg/m^2^)	30.2 ± 5.6	29.8 ± 6.2	0.781
Gestational age (week)	39.6 ± 0.8	39.5 ± 0.9	0.436

Data are presented as mean ± SD.

**TABLE 2:** Maternal and neonatal outcomes.

Index	Ondansetron group (*n* = 40)	Control group (*n* = 40)	*p*-value
Induction-to-delivery interval (min)	21.4 ± 2.7	20.6 ± 2.9	0.252
Total volumes of fluid given (mL)	732 ± 50	735 ± 63	0.860
Use of vasoconstrictor (n)	2 (5)	10 (25)	0.012*
Highest levels of sensory block [T4/T6] (n)	22/18	21/19	0.823
Lowest mean arterial pressure (mmHg)	71.6 ± 4.9	68.8 ± 6.4	0.028*
1-min Apgar score	9.1 ± 0.6	9.0 ± 0.6	0.468
5-min Apgar score	9.4 ± 0.5	9.5 ± 0.6	0.846
Umbilical artery pH	7.31 ± 0.03	7.28 ± 0.04	0.002*

Data are presented as mean ± SD or number (percent); *p < 0.05.

**FIGURE 2:**
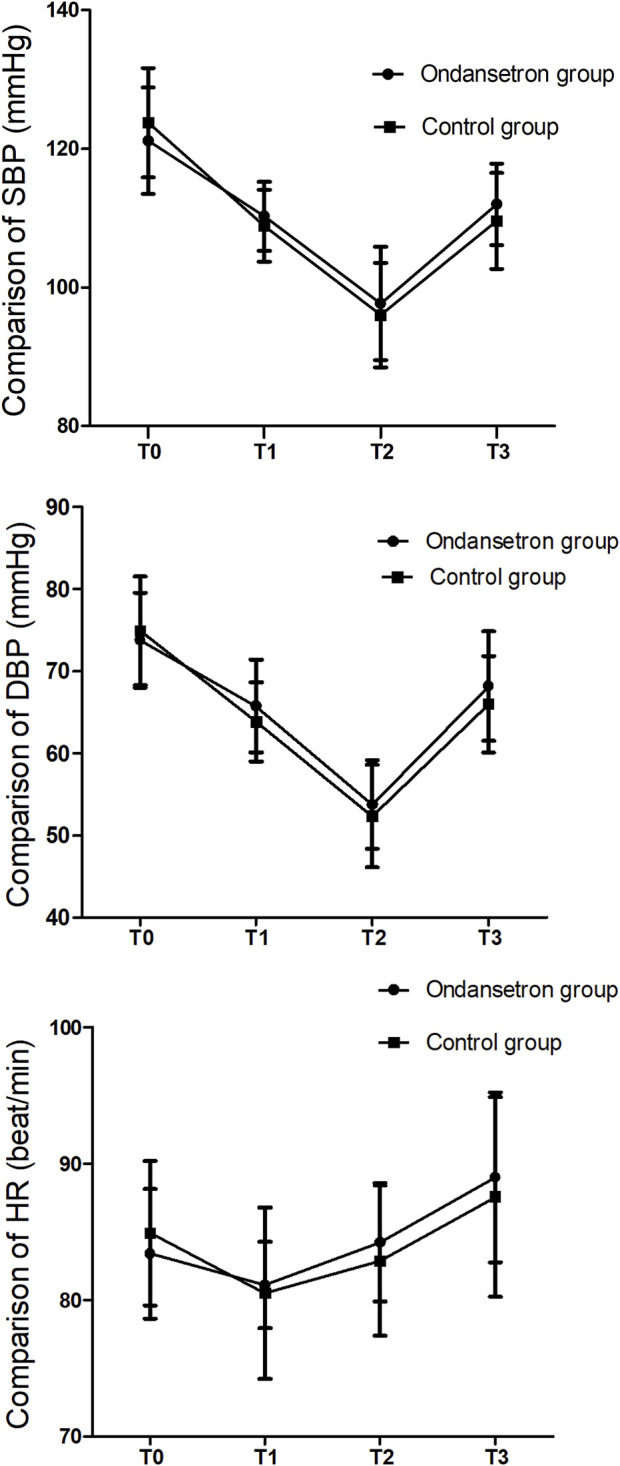
Comparison of the hemodynamics.

The blood pressure and HR did not change significantly before and after drug administration. The SBP, DBP, and HR were similar at each time point after spinal anesthesia; there were no significant differences between the two groups (Figure 2).

The incidence of supine hypotensive syndrome, nausea, and vomiting was significantly lower in the ondansetron group than the control group (2.5% vs. 20%, *p* = 0.029; 2.5% vs. 22.5%, *p* = 0.007, respectively), but there were no statistical significances in terms of hypotension, bradycardia, respiratory depression, and fatal acidosis between the two groups (*p* > 0.05) (Table 3).

**TABLE 3:** Adverse events of the women and neonates.

Index	Ondansetron group (*n* = 40)	Control group (*n* = 40)	*p-*value
Supine hypotensive syndrome	1 (2.5)	8 (20)	0.029*
Hypotension	16 (4 (0)	18 (45)	0.651
Bradycardia	2 (5)	3 (7.5)	0.999
Nausea and vomiting	1 (2.5)	9 (22.5)	0.007*
Respiratory depression	0 (0)	0 (0)	0.999
Fetal acidosis	0 (0)	0 (0)	0.999

Data are shown as number (%) compared with the control group; *p < 0.05.

## 4 Discussion

In this study, we found that ondansetron can effectively prevent supine hypotensive syndrome, reduce the incidence of nausea, vomiting, and vasoconstrictor drug use, and improve neonatal umbilical artery pH during spinal anesthesia for cesarean section.

In the present study, the blood pressure and heart rate 5 min after ondansetron administration had no obvious changes, which indicated that ondansetron had no effects on maternal hemodynamics before and after spinal anesthesia. In our study, only one case of supine hypotensive syndrome was observed; almost all parturients who were administered ondansetron were able to tolerate lying supine during spinal anesthesia without experiencing any symptoms except hypotension. The finding demonstrated that ondansetron can effectively prevent supine hypotensive syndrome during spinal anesthesia for cesarean section. The relevant reasons are as follows: first, ondansetron is a serotonin receptor antagonist; it can inhibit the Bezold–Jarisch reflex and reduce spinal anesthesia-induced vasodilation. Second, ondansetron may cause a dose-dependent increase in blood pressure by acting on serotonin receptors located at the medial septum/vertical limb of the diagonal band complex (Urzedo-Rodrigues et al., 2011). Finally, animal studies indicate that serotonin pathways in the rat brain are a complicated and multifactorial system that regulates blood pressure (Fregoneze et al., 2011); thus, the peripheral and central mechanisms can be involved. Our study indicated that the minimal mean arterial pressure was higher in the ondansetron group than the control group (71.4 ± 4.9 vs. 68.8 ± 6.4 mmHg, *p* = 0.028). Ondansetron can inhibit the Bezold–Jarisch reflex and mitigate hypotension after spinal anesthesia in the event of a further drop in blood pressure. Ondansetron can attenuate serotonin-induced vasodilation and lead to a slight increase in blood pressure, so ondansetron could not decrease the incidence of maternal hypotension during spinal anesthesia for cesarean section. The present study also showed that ondansetron could significantly reduce the incidence of maternal nausea and vomiting. There is a controversial issue about ondansetron reducing the incidence of hypotension in a cesarean section. Wang et al. (2014) reported that intravenous ondansetron (4 mg) prior to spinal anesthesia can reduce the incidence of hypotension in cesarean sections by 30% vs. 60%, which is contrary to our findings. The reason may be related to the small sample size. However, some studies indicated that ondansetron could not reduce the incidence of hypotension in parturient women (Owczuk et al., 2008; Marciniak et al., 2015), which is in agreement with our findings. Ondansetron could attenuate the fall of systolic and mean blood pressure but did not decrease the incidence of hypotension induced by spinal anesthesia. During cesarean sections, the occurrence of nausea and vomiting was mainly associated with hypotension. Hypotension stimulates the central nervous system, which leads to the occurrence of nausea and vomiting. Moreover, ondansetron reduces the occurrence of nausea and vomiting by inhibiting the emetic center in the brain. In this study, we found a strange phenomenon: the parturient women who were administered ondansetron were able to tolerate hypotension better and did not feel any discomfort. Because of ethical issues, the vasoconstrictor drugs were given when mean arterial pressure was <60 mmHg (the lowest blood pressure that an important organ can tolerate) in this study, thus failing to investigate the minimum mean arterial pressure that can be tolerated.

In this study, the umbilical artery pH was higher in the ondansetron group than the control group, but no fetal acidosis (umbilical artery pH less than 7.2) was observed. Umbilical artery pH is a sensitive index that reflects fetal asphyxia. Umbilical artery pH is mainly associated with fetal perfusion. Ondansetron plays an important role in increasing fetal perfusion by inhibiting the Bezold–Jarisch reflex and reducing serotonin-induced vasodilation after spinal anesthesia. The 5-min Apgar scores of the neonates were all greater than 9 in both groups, which showed that ondansetron had no adverse effect on the neonates. Pasternak et al. (2013) reported that more than 600,000 pregnant women treated with ondansetron had no adverse effects on the neonates. In addition, Einarson et al. (2004) reported that ondansetron administered during pregnancy was not associated with an increased risk for major birth defects above the baseline level. These findings were consistent with the results of our study.

### 4.1 Limitations

Genetic polymorphism, baseline sympathovagal balance, and environmental and individual factors may influence spinal anesthesia-induced hypotension during elective cesarean section, which increases outcome variability (Yu et al., 2021). Moreover, research with a large sample size is needed to explore the safety of the serotonin receptor antagonist in fetuses.

## 5 Conclusion

Ondansetron can effectively prevent supine hypotensive syndrome, reduce the incidence of nausea, vomiting, and vasoconstrictor drug usage, and improve neonatal umbilical arterial pH during spinal anesthesia for cesarean section.

## Data Availability

The raw data supporting the conclusion of this article will be made available by the authors, without undue reservation.
